# Crystal structure of the [(THF)Cs(μ-η^5^:η^5^-Cp′)_3_Yb]_*n*_ oligomer

**DOI:** 10.1107/S2056989020008051

**Published:** 2020-06-23

**Authors:** Daniel N. Huh, Joseph W. Ziller, William J. Evans

**Affiliations:** a Department of Chemistry, University of California, Irvine, California, 92697, USA

**Keywords:** lanthanide, caesium, cyclo­penta­dienide, oligomer, crystal structure

## Abstract

The green compound poly[(tetra­hydro­furan)­tris­[μ-η^5^:η^5^-1-(tri­methyl­sil­yl)cyclo­penta­dien­yl]caesium(I)ytterbium(II)], [CsYb(C_8_H_13_Si)_3_(C_4_H_8_O)]_*n*_ or [(THF)Cs(μ-η^5^:η^5^-Cp′)_3_Yb^II^]_*n*_ was synthesized by reduction of a red THF solution of (C_5_H_4_SiMe_3_)_3_Yb^III^ with excess Cs metal and identified by X-ray diffraction. The compound crystallizes as a two-dimensional array of hexa­gons with alternating Cs^I^ and Yb^II^ ions at the vertices and cyclo­penta­dienyl groups bridging each edge. This, based off the six-electron cyclo­penta­dienyl rings occupying three coordination positions, gives a formally nine-coordinate tris­(cyclo­penta­dien­yl) coordination environment to Yb and the Cs is ten-coordinate due to the three cyclo­penta­dienyl rings and a coordinated mol­ecule of THF. The complex comprises layers of Cs_3_Yb_3_ hexa­gons with THF ligands and Me_3_Si groups in between the layers. The Yb—C metrical parameters are consistent with a 4*f* ^14^ Yb^II^ electron configuration.

## Abstract 2

Reduction of (C_5_H_4_SiMe_3_)_3_Yb^III^ in THF using excess Cs metal forms the oligomeric complex [(THF)Cs(μ-η^5^:η^5^-Cp′)_3_Yb^II^]*_n_.* The complex has hexa­gonal layers of Cs_3_Yb_3_ with THF ligands and Me_3_Si groups in between the layers.

## Chemical context   

**Figure 1:**
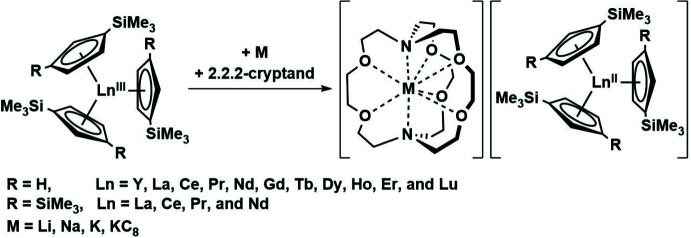
Synthesis of (Cp^*x*^_3_ *Ln* ^II^)^1−^ complexes by alkali metal reduction of Cp^*x*^_3_ *Ln* ^III^ precursors; Cp^*x*^ = C_5_H_4_SiMe_3_, C_5_H_3_(SiMe_3_)_2_.

The new +2 oxidation states for the rare-earth metals Y, La, Ce, Pr, Gd, Tb, Ho, Er, and Lu were recently discovered by reduction of Cp^*x*^_3_ *Ln* (Cp^*x*^ = C_5_H_4_SiMe_3_, C_5_H_3_(SiMe_3_)_2_; *Ln* = rare-earth metal) using alkali metal reductants Li, Na, K, and KC_8_ (Fig. 1 ▸) (Hitchcock *et al.*, 2008 ▸; MacDonald *et al.*, 2013 ▸; Fieser *et al.*, 2015 ▸; Evans, 2016 ▸; Palumbo *et al.*, 2018 ▸). In each of these cases, 2.2.2-cryptand was added in these reactions to encapsulate the alkali metal. It was thought that chelating agents were necessary to sequester the alkali metal to prevent inter­actions with cyclo­penta­dienide ligands and subsequent ligand dissociation leading to product decomposition. This idea was challenged by examining reduction reactions of Cp′′_3_ *M* (Cp′′ = C_5_H_3_(SiMe_3_)_2_; *M* = La, Ce, U) with Li and Cs in the absence of chelating agents (Huh *et al.*, 2018 ▸). The reaction resulted in the isolation of the first chelate-free synthesis of La^II^, Ce^II^, and U^II^ complexes. The [Li(THF)_4_]^1+^ cation of the Li salts in these chelate-free **M** ^II^ complexes were well-separated from the (Cp′′_3_ *M*)^1−^ anion. However, the Cs reductions yielded polymeric complexes of general formula [Cp′′*M*(μ-Cp′′)_2_Cs(THF)_2_]_*n*_ where the Cs cation has coord­in­ated THF and cyclo­penta­dienide ligands. Attempts to extend this chemistry to smaller rare-earth metals by reduction of Cp′_3_ *Ln* (Cp′ = C_5_H_4_SiMe_3_; *Ln* = Y, Tb, Dy) showed evidence of *Ln* ^II^ in solution; however, the reduction products were highly unstable and decomposed even at 238 K.
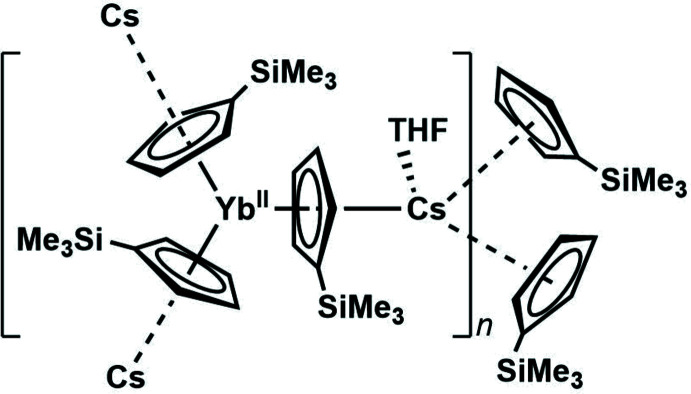


**Figure 2:**
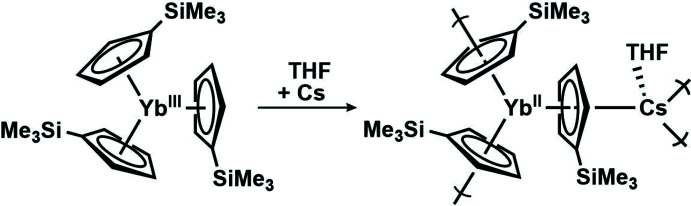
Synthesis of [(THF)Cs(μ-η^5^:η^5^-Cp′)_3_Yb^II^]_*n*_, **1**, by caesium metal reduction of the Cp′_3_Yb^III^ precursor.

In this study, we were inter­ested in examining the reduction of Cp′_3_Yb^III^ with Cs metal. Unlike Y^II^, Tb^II^, and Dy^II^ ions, Yb^II^ complexes are more easily obtainable, as reflected by their less negative reduction potentials (Morss, 1976 ▸). A crystal containing the oligomeric compound; [(THF)Cs(μ-η^5^:η^5^-Cp′)_3_Yb]_*n*_, **1** (Cp′ = C_5_H_4_SiMe_3_) was isolated by reduction of the Cp′_3_Yb^III^ complex (Fieser *et al.*, 2015 ▸) in THF using Cs metal (Figs. 2 ▸ and 3 ▸).

## Structural commentary   

All three Cp′ rings remain coordinated to the Yb metal center after reduction and are coordinated in a trigonal–planar fashion. The Yb atom is within 0.107 Å of the plane of the three ring centroids. Each ring bridges Yb to Cs, which also is surrounded by three cyclo­penta­dienyl ligands as well as a coordinated mol­ecule of THF. The three ring centroids and the oxygen of THF are arranged in a pseudo-tetra­hedral geometry around Cs with a calculated four-coordinate Cs τ′_4_ value of 0.76 (τ′_4_ = 1 for tetra­hedral; τ′_4_ = 0 for square planar; Rosiak *et al.*, 2018 ▸). The Cs metal center has a pseudo-tetra­hedral geometry with Cp′(centroid)⋯Cs⋯Cp′(centroid) angles of 109.0, 114.3, and 121.4° and Cp′(centroid)⋯Cs⋯O(THF) angles of 88.8, 94.1, and 127.8°.

**Figure 4:**
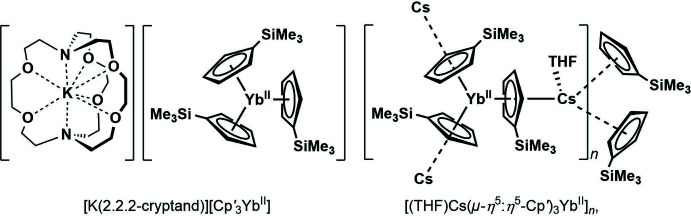
*CHEMDRAW* (Mills, 2006 ▸) representation of [K(2.2.2-cryptand)][Cp′_3_Yb^II^] (left) and [(THF)Cs(μ-η^5^:η^5^-Cp′)_3_Yb^II^]_*n*_, **1**, (right).

The bond distances and angles in **1** are summarized in Table 1 ▸. The range of 2.504 (1)–2.513 (2) Å Cp′(centroid)⋯Yb bond distances in **1** is the same as that in the complex [K(crypt)][Cp′_3_Yb^II^] (crypt = 2.2.2-cryptand), which was fully characterized as a 4*f* ^14^ Yb^II^ complex, Table 2 ▸ and Fig. 4 ▸. In Cp_3_ *Ln* reduction chemistry, the difference in *Ln*⋯Cp(centroid) distances between the *Ln* ^III^ and *Ln* ^II^ complexes provides important information on the electronic configuration of the lanthanide ion (Evans, 2016 ▸). Differences in *Ln⋯*Cp(centroid) distances for reduction of 4*f* ^*n*^ *Ln* ^III^ ions to 4*f* ^*n*+1^ *Ln* ^II^ ions range from 0.1 to 0.2 Å (Fieser *et al.*, 2015 ▸). In this study, the difference of 0.14 Å in the *Ln*⋯Cp(centroid) distance is characteristic of a 4*f* ^13^ Yb^III^ reduction to a 4*f* ^14^ Yb^II^ ion. In contrast, *Ln* ^II^ ions with 4*f^*n*^*5*d* ^1^ configurations where the additional electron populates a *d*-orbital instead of the an *f*-orbital have differences of only 0.02–0.05 Å (Evans, 2016 ▸).

## Supra­molecular features   

**Figure 5:**
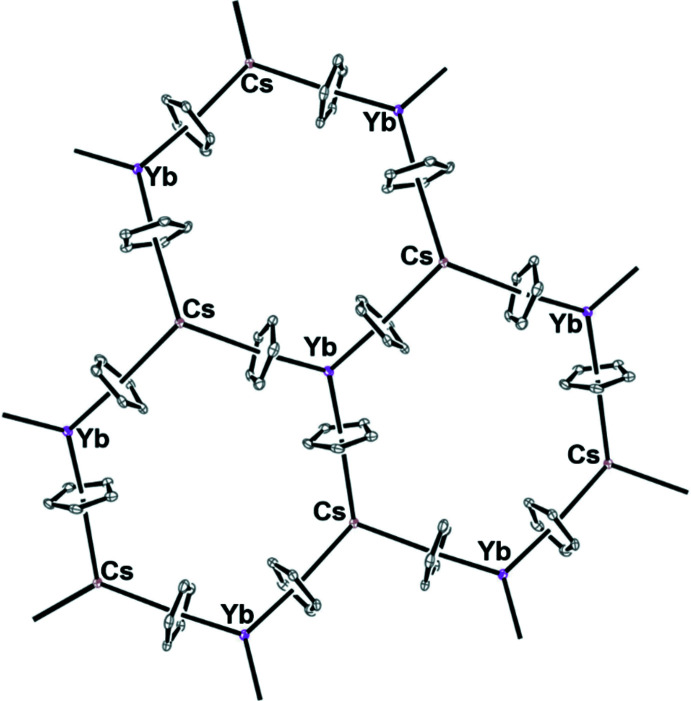
Top view of the extended structure of [(THF)Cs(μ-η^5^:η^5^-Cp′)_3_Yb]_*n*_, **1**, with the SiMe_3_ substituent of the C_5_H_4_SiMe_3_ group and the THF attached to Cs removed for clarity.

In **1**, all of the cyclo­penta­dienyl ligands are bridging. The threefold symmetry of three bridging Cp′ ligands on each metal generates a hexa­gonal pattern as shown in Fig. 5 ▸. The Yb⋯Cp′(centroid)⋯Cs angles are 172.5–176.7° such that each side of the hexa­gon is nearly linear. The 112.4–117.3° Yb⋯Cs⋯Yb angles are smaller than the 120.8–125.6° Cs⋯Yb⋯Cs angles, which makes the hexa­gon slightly irregular. This could be of inter­est to quantum scientists trying to make thin-film layers of magnetic materials since the hexa­gonal pattern could lead to spin frustration with a paramagnetic lanthanide.

**Figure 6:**
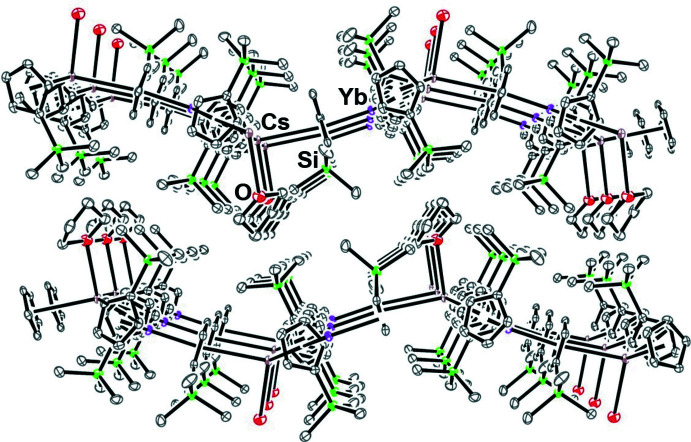
Side view of the extended structure of [(THF)Cs(μ-η^5^:η^5^-Cp′)_3_Yb]_*n*_, **1**. Magenta, Yb; brown, Cs; green, Si; red, O.

The side view of these layers in Fig. 6 ▸ shows how the space in between them is filled with THF and Me_3_Si substituent groups. The 116.6–122.8° Cp′(centroid)⋯Yb⋯Cp′(centroid) and 109.0–121.4° Cp′(centroid)⋯Cs⋯Cp′(centroid) angles generate the undulation of the hexa­gons shown in Fig. 6 ▸.

## Database survey   

The 3.159 (1), 3.197 (1), and 3.268 (2) Å Cs⋯Cp′(centroid) distances in **1** are shorter than the 3.278 and 3.435 Å Cs⋯Cp′′(centroid) distances in [(THF)_2_Cs][(μ-η^5^:η^5^-Cp′′)_2_U^II^(η^5^-Cp′′)]_*n*_, (Huh *et al.*, 2018 ▸), the 3.396 Å Cs⋯C_5_H_5_(centroid) distances in {[(Me_3_Si)_2_NCs]_2_·[(C_5_H_5_)_2_Fe)] 0.5·(C_6_H_5_Me)}_*n*_, (Morris *et al.*, 2007 ▸) and the 3.337 Å Cs⋯C_5_Me_5_(centroid) distances in [(THF)_2_Cs(μ_3_-O)_3_{[Ti(C_5_Me_5_)]_3_-(μ_3_-CCH_2_)}] (González-del Moral *et al.*, 2005 ▸). The 3.095 (3) Å Cs—O(THF) bond distance is consistent with the Cs—O(THF) distances of 3.081 (7) to 3.119 (8) Å in [(THF)_2_Cs][(μ-η^5^:η^5^-Cp′′)_2_U^II^(η^5^-Cp′′)]_*n*_ (Huh *et al.*, 2018 ▸) and 3.034 (9)–3.06 (1) Å in [(THF)_2_Cs(μ_3_-O)_3_{[Ti(C_5_Me_5_)]_3_(μ_3_-CCH_2_)}] (González-del Moral *et al.*, 2005 ▸).

The extended structure of **1** differs from that of the [(THF)_2_Cs][(μ-η^5^:η^5^-Cp′′)_2_ *M* ^II^(η^5^-Cp′′)]_*n*_, complexes (*M* = La, U), which comprise zigzag chains of –*M*–(μ-Cp′′)–Cs–(μ-Cp′′)– repeat units with a terminal Cp′′ attached to *M* and two terminal THF ligands attached to Cs (Huh *et al.*, 2018 ▸). These were obtained by reduction of Cp′′_3_ *M* ^III^ compounds with Cs in THF. In those structures, La and U have a trigonal–planar tris­(cyclo­penta­dien­yl) coordination like Yb in **1**, but the Cs is coordinated by only two cyclo­penta­dienyl ligands to give a bent metallocene Cp′′_2_Cs(THF)_2_ sub-structure with these larger rings.

A survey of the Cambridge Structural Database (CSD, version 5.41, March 2020; Groom *et al.*, 2016 ▸) also revealed four oligomeric complexes containing Yb–Cp^*x*^ moieties with various types of cyclo­penta­dienyl rings (Cp^*x*^): [Na(μ-η^5^:η^5^-C_5_H_5_)_3_Yb^II^]_*n*_ (Apostolidis *et al.*, 1997 ▸), [Na(μ-η^5^:η^5^-Cp′′)_2_Yb^II^_2_(μ-η^5^:η^5^-Cp′′)_2_]_*n*_ (Voskoboynikov *et al.*, 1997 ▸), [(C_5_Me_5_)Yb(μ-I)(μ-η^5^:η^5^-C_5_Me_5_)Yb(C_5_Me_5_)]_*n*_ (Evans *et al.*, 2006 ▸) and [Yb(μ-η^5^:η^5^-C_5_H_5_)(Ph_2_Pz)(THF)]_*n*_ (Ph_2_Pz = 3,5–di­phenyl­pyrazolate) (Ali *et al.*, 2018 ▸). The [Na(μ-η^5^:η^5^-C_5_H_5_)_3_Yb^II^]_*n*_ (Apostolidis *et al.*, 1997 ▸) complex adopts a hexa­gonal net extended structure similar to that in **1** except the alkali metal does not have a coordinated solvent. The structure of [Na(μ-η^5^:η^5^-Cp^*t*Bu^)_3_Sm^II^] is similar (Bel’sky *et al.*, 1990 ▸). Three oligomeric complexes containing Cs–cyclo­penta­dienyl moieties have previously been reported: [(THF)_2_Cs][(μ-η^5^:η^5^-Cp′′)_2_U^II^(η^5^-Cp′′)]_*n*_ (Huh *et al.*, 2018 ▸), {[(Me_3_Si)_2_NCs]_2_[(C_5_H_5_)_2_Fe)]·0.5(C_6_H_5_Me)}_*n*_ (Morris *et al.*, 2007 ▸) and [(THF)_2_Cs(μ_3_-O)_3_{[Ti(C_5_Me_5_)]_3_(μ_3_-CCH_2_)}] (Gon­zález-del Moral *et al.*, 2005 ▸). An oligomeric, base-free Li–Cp′ compound was also previously reported in the literature, [(μ-η^5^:η^5^-Cp′)Li]_*n*_ (Evans *et al.*, 1992 ▸).

## Synthesis and crystallization   

In an argon-filled glovebox, addition of a red solution of Cp′_3_Yb (50 mg, 0.085 mmol) in THF (2 mL) to excess Cs as a smear produced a green solution. This was stirred for 15 min at room temperature and then layered at the bottom of a vial below an Et_2_O (10 mL) layer for crystallization at −35°C. After 1 d, X-ray quality dark-green crystals of [(THF)Cs(μ-η^5^:η^5^-Cp′)_3_Yb^II^]_*n*_ were isolated. A small number of crystals were obtained and used for crystallographic analysis. Too little sample was available for other characterization.

## Refinement   

Crystal data and structure refinement for [(THF)Cs(μ-η^5^:η^5^-Cp′)_3_Yb^II^]_*n*_, **1** are summarized in Table 3 ▸. Hydrogen atoms were included using a riding model with *U*_iso_(H) values of 1.2*U*_eq_(C) for CH_2_ and aromatic hydrogens and 1.5*U*_eq_(C) for CH_3_ hydrogens with C—H distances of 0.99 (CH_2_), 0.95 (aromatic), and 0.98 Å (CH_3_).

## Supplementary Material

Crystal structure: contains datablock(s) I. DOI: 10.1107/S2056989020008051/zl2785sup1.cif

Structure factors: contains datablock(s) I. DOI: 10.1107/S2056989020008051/zl2785Isup2.hkl

CCDC reference: 2010185

Additional supporting information:  crystallographic information; 3D view; checkCIF report

## Figures and Tables

**Figure 3:**
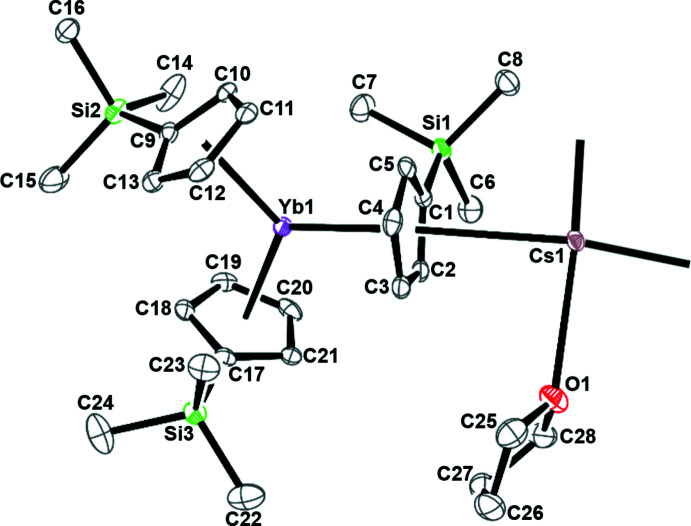
*ORTEP* representation of an asymmetric unit of [(THF)Cs(μ-η^5^:η^5^-Cp′)_3_Yb]_*n*_, **1**, with probability ellipsoids drawn at the 50% probability level. Hydrogen atoms were omitted for clarity.

**Table 1:** Selected bond distances and angles for [(THF)Cs(μ-η^5^:η^5^-Cp′)_3_Yb]_*n*_, **1** Centroid1, centroid2, and centroid3 are the centroids of the Cp rings connected to Si1, Si2, and Si3, respectively.

Yb1⋯centroid1	2.510 (1)
Yb1⋯centroid2	2.513 (2)
Yb1⋯centroid3	2.504 (1)
Cs1⋯centroid1	3.197 (1)
Cs1⋯centroid2	3.268 (2)
Cs1⋯centroid3	3.159 (1)
Cs1—O1	3.095 (3)
	
centroid1—Yb1⋯centroid2	120.1
centroid1—Yb1⋯centroid3	116.6
centroid2—Yb1⋯centroid3	122.8
centroid1—Cs1⋯centroid2	121.4
centroid1—Cs1⋯centroid3	109.0
centroid2—Cs1⋯centroid3	114.3
Yb1⋯centroid1⋯Cs1	175.3
Yb1⋯centroid2⋯Cs1	172.3
Yb1⋯centroid3⋯Cs1	176.7
centroid1⋯Cs1⋯O1	88.8
centroid2⋯Cs1⋯O1	94.1
centroid3⋯Cs1⋯O1	127.8

**Table 2:** Bond distance (Å) ranges for Yb⋯Cp′(centroid) and bond angle (°) ranges for Cp′(centroid)⋯Yb⋯Cp′(centroid) in Cp′_3_Yb (Fieser *et al.*, 2015 ▸), [K(crypt)][Cp′_3_Yb] (Fieser *et al.*, 2015 ▸), and [(THF)Cs(μ-η^5^:η^5^-Cp′)_3_Yb]_*n*_

	Cp′_3_Yb	[K(crypt)][Cp′_3_Yb]	**1**
Yb⋯Cp′(centroid)	2.363–2.368	2.503–2.513	2.504 (1)–2.513 (2)
Cs⋯Cp′(centroid)			3.159 (1)–3.268 (2)
Cp′⋯Yb⋯Cp′	118.85–120.55	118.10–122.93	116.64–122.76
Cp′⋯Cs⋯Cp′			109.0–121.4

**Table 3:** Experimental details

Crystal data	
Chemical formula	[CsYb(C_8_H_13_Si)_3_(C_4_H_8_O)]
*M*_r_	789.87
Crystal system, space group	Monoclinic, *P*2_1_/*n*
Temperature (K)	88
*a*, *b*, *c* (Å)	9.4401 (4), 16.8718 (8), 21.0246 (10)
β (°)	92.0668 (6)
*V* (Å^3^)	3346.4 (3)
*Z*	4
Radiation type	Mo *K*α
μ (mm^−1^)	3.99
Crystal size (mm)	0.15 × 0.09 × 0.08

Data collection	
Diffractometer	Bruker SMART APEXII CCD
Absorption correction	Multi-scan (*SADABS*; Bruker, 2014 ▸)
*T*_min_, *T*_max_	0.374, 0.432
No. of measured, independent and observed [*I* > 2σ(*I*)] reflections	40586, 8223, 6580
*R*_int_	0.055
(sin θ/λ)_max_ (Å^−1^)	0.667

Refinement	
*R*[*F* ^2^ > 2σ(*F* ^2^)], *wR*(*F* ^2^), *S*	0.032, 0.056, 1.02
No. of reflections	8223
No. of parameters	316
H-atom treatment	H-atom parameters constrained
Δρ_max_, Δρ_min_ (e Å^−3^)	1.14, −0.62

Computer programs: *APEX2* (Bruker, 2014 ▸), *SAINT* (Bruker, 2013 ▸), *SHELXT2014/4* (Sheldrick, 2015*a* ▸), *SHELXL2014/7* (Sheldrick, 2015*b* ▸) and *SHELXTL* (Sheldrick, 2008 ▸).

## supplementary crystallographic information

### Crystal data

**Table:**

[CsYb(C_8_H_13_Si)_3_(C_4_H_8_O)]	*F*(000) = 1560
*M**_r_* = 789.87	*D*_x_ = 1.568 Mg m^−^^3^
Monoclinic, *P*2_1_/*n*	Mo *K*α radiation, λ = 0.71073 Å
*a* = 9.4401 (4) Å	Cell parameters from 9869 reflections
*b* = 16.8718 (8) Å	θ = 2.3–28.5°
*c* = 21.0246 (10) Å	µ = 3.99 mm^−^^1^
β = 92.0668 (6)°	*T* = 88 K
*V* = 3346.4 (3) Å^3^	Prism, green
*Z* = 4	0.15 × 0.09 × 0.08 mm

### Data collection

**Table:**

Bruker SMART APEXII CCD diffractometer	6580 reflections with *I* > 2σ(*I*)
Radiation source: fine-focus sealed tube	*R*_int_ = 0.055
φ and ω scans	θ_max_ = 28.3°, θ_min_ = 1.6°
Absorption correction: multi-scan (SADABS; Bruker, 2014)	*h* = −12→12
*T*_min_ = 0.374, *T*_max_ = 0.432	*k* = −22→22
40586 measured reflections	*l* = −27→27
8223 independent reflections	

### Refinement

**Table:**

Refinement on *F*^2^	Primary atom site location: structure-invariant direct methods
Least-squares matrix: full	Secondary atom site location: difference Fourier map
*R*[*F*^2^ > 2σ(*F*^2^)] = 0.032	Hydrogen site location: inferred from neighbouring sites
*wR*(*F*^2^) = 0.056	H-atom parameters constrained
*S* = 1.02	*w* = 1/[σ^2^(*F*_o_^2^) + (0.0229*P*)^2^ + 0.1327*P*] where *P* = (*F*_o_^2^ + 2*F*_c_^2^)/3
8223 reflections	(Δ/σ)_max_ = 0.001
316 parameters	Δρ_max_ = 1.14 e Å^−^^3^
0 restraints	Δρ_min_ = −0.62 e Å^−^^3^

### Special details

**Table:**

Geometry. All esds (except the esd in the dihedral angle between two l.s. planes) are estimated using the full covariance matrix. The cell esds are taken into account individually in the estimation of esds in distances, angles and torsion angles; correlations between esds in cell parameters are only used when they are defined by crystal symmetry. An approximate (isotropic) treatment of cell esds is used for estimating esds involving l.s. planes.
Refinement. A green crystal of approximate dimensions 0.079 x 0.086 x 0.148 mm was mounted in a cryoloop and transferred to a Bruker SMART APEX II diffractometer. The APEX2 program package was used to determine the unit-cell parameters and for data collection (90 sec/frame scan time for a sphere of diffraction data). The raw frame data was processed using SAINT and SADABS to yield the reflection data file. Subsequent calculations were carried out using the SHELXTL program. The diffraction symmetry was 2/m and the systematic absences were consistent with the monoclinic space group P21/n that was later determined to be correct. The structure was solved by dual space methods and refined on F2 by full-matrix least-squares techniques. The analytical scattering factors for neutral atoms were used throughout the analysis. Hydrogen atoms were included using a riding model. The structure is polymeric. Least-squares analysis yielded wR2 = 0.0562 and Goof = 1.017 for 316 variables refined against 8223 data (0.75 Å), R1 = 0.0315 for those 6580 data with I > 2.0sigma(I).

### Fractional atomic coordinates and isotropic or equivalent isotropic displacement parameters (Å^2^)

**Table:**

	*x*	*y*	*z*	*U*_iso_*/*U*_eq_	
Yb1	0.48657 (2)	0.44122 (2)	0.25290 (2)	0.01341 (4)	
Cs1	0.99437 (2)	0.27157 (2)	0.31696 (2)	0.01493 (5)	
Si1	0.69834 (10)	0.29048 (6)	0.15029 (5)	0.0184 (2)	
Si2	0.23952 (11)	0.57037 (6)	0.11663 (5)	0.0204 (2)	
Si3	0.25791 (10)	0.45182 (6)	0.40988 (5)	0.0187 (2)	
O1	0.8795 (3)	0.24593 (16)	0.45145 (12)	0.0285 (6)	
C1	0.7108 (3)	0.33895 (19)	0.22942 (16)	0.0161 (7)	
C2	0.6551 (3)	0.3115 (2)	0.28743 (17)	0.0174 (8)	
H2A	0.6070	0.2626	0.2929	0.021*	
C3	0.6826 (3)	0.3680 (2)	0.33517 (17)	0.0196 (8)	
H3A	0.6554	0.3643	0.3781	0.024*	
C4	0.7575 (4)	0.4310 (2)	0.30831 (17)	0.0194 (8)	
H4A	0.7902	0.4774	0.3298	0.023*	
C5	0.7751 (3)	0.4129 (2)	0.24430 (17)	0.0184 (8)	
H5A	0.8231	0.4453	0.2150	0.022*	
C6	0.6270 (4)	0.1878 (2)	0.15741 (18)	0.0257 (9)	
H6A	0.6848	0.1582	0.1890	0.039*	
H6B	0.6299	0.1611	0.1161	0.039*	
H6C	0.5288	0.1902	0.1709	0.039*	
C7	0.5764 (4)	0.3487 (2)	0.09635 (17)	0.0276 (9)	
H7A	0.6159	0.4017	0.0900	0.041*	
H7B	0.4836	0.3533	0.1155	0.041*	
H7C	0.5656	0.3216	0.0552	0.041*	
C8	0.8767 (4)	0.2878 (2)	0.11484 (18)	0.0263 (9)	
H8A	0.9435	0.2601	0.1438	0.039*	
H8B	0.9099	0.3421	0.1081	0.039*	
H8C	0.8702	0.2599	0.0740	0.039*	
C9	0.3846 (3)	0.57009 (19)	0.17806 (16)	0.0162 (7)	
C10	0.5320 (4)	0.56244 (19)	0.16482 (17)	0.0173 (7)	
H10A	0.5679	0.5462	0.1253	0.021*	
C11	0.6148 (4)	0.58254 (19)	0.21886 (17)	0.0171 (8)	
H11A	0.7154	0.5833	0.2220	0.021*	
C12	0.5224 (4)	0.60152 (19)	0.26788 (17)	0.0190 (8)	
H12A	0.5497	0.6163	0.3102	0.023*	
C13	0.3823 (4)	0.59465 (19)	0.24280 (16)	0.0180 (8)	
H13A	0.2992	0.6048	0.2656	0.022*	
C14	0.2553 (5)	0.4858 (2)	0.0598 (2)	0.0380 (11)	
H14A	0.2394	0.4358	0.0822	0.057*	
H14B	0.3503	0.4856	0.0425	0.057*	
H14C	0.1843	0.4916	0.0249	0.057*	
C15	0.0612 (4)	0.5700 (3)	0.1518 (2)	0.0379 (11)	
H15A	0.0433	0.5178	0.1705	0.057*	
H15B	−0.0113	0.5811	0.1185	0.057*	
H15C	0.0577	0.6107	0.1849	0.057*	
C16	0.2543 (4)	0.6620 (2)	0.06760 (17)	0.0230 (8)	
H16A	0.3509	0.6665	0.0524	0.034*	
H16B	0.2329	0.7085	0.0936	0.034*	
H16C	0.1869	0.6593	0.0311	0.034*	
C17	0.2651 (3)	0.4025 (2)	0.33118 (16)	0.0151 (7)	
C18	0.1959 (3)	0.42821 (19)	0.27347 (17)	0.0169 (7)	
H18A	0.1408	0.4751	0.2686	0.020*	
C19	0.2216 (3)	0.3741 (2)	0.22535 (17)	0.0187 (8)	
H19A	0.1879	0.3780	0.1823	0.022*	
C20	0.3061 (4)	0.3127 (2)	0.25131 (17)	0.0206 (8)	
H20A	0.3395	0.2678	0.2291	0.025*	
C21	0.3320 (3)	0.3300 (2)	0.31617 (17)	0.0173 (8)	
H21A	0.3858	0.2981	0.3453	0.021*	
C22	0.2748 (5)	0.3775 (2)	0.47533 (18)	0.0346 (10)	
H22A	0.1956	0.3400	0.4718	0.052*	
H22B	0.2732	0.4047	0.5165	0.052*	
H22C	0.3644	0.3487	0.4721	0.052*	
C23	0.4026 (4)	0.5264 (2)	0.42253 (18)	0.0280 (9)	
H23A	0.3884	0.5702	0.3923	0.042*	
H23B	0.4944	0.5012	0.4158	0.042*	
H23C	0.4008	0.5469	0.4661	0.042*	
C24	0.0843 (4)	0.5039 (3)	0.4147 (2)	0.0399 (11)	
H24A	0.0071	0.4652	0.4106	0.060*	
H24B	0.0748	0.5429	0.3803	0.060*	
H24C	0.0798	0.5309	0.4559	0.060*	
C25	0.8427 (4)	0.3003 (2)	0.50059 (19)	0.0306 (9)	
H25A	0.8093	0.3512	0.4820	0.037*	
H25B	0.9255	0.3108	0.5296	0.037*	
C26	0.7248 (4)	0.2603 (2)	0.53629 (19)	0.0338 (10)	
H26A	0.7634	0.2246	0.5700	0.041*	
H26B	0.6615	0.2998	0.5555	0.041*	
C27	0.6489 (4)	0.2145 (2)	0.4835 (2)	0.0315 (10)	
H27A	0.5959	0.1690	0.5005	0.038*	
H27B	0.5827	0.2489	0.4585	0.038*	
C28	0.7700 (4)	0.1873 (2)	0.44422 (18)	0.0278 (9)	
H28A	0.8052	0.1351	0.4593	0.033*	
H28B	0.7388	0.1823	0.3989	0.033*	

### Atomic displacement parameters (Å^2^)

**Table:**

	*U*^11^	*U*^22^	*U*^33^	*U*^12^	*U*^13^	*U*^23^
Yb1	0.01020 (7)	0.01233 (7)	0.01766 (8)	−0.00136 (6)	−0.00014 (6)	0.00251 (6)
Cs1	0.01034 (10)	0.01249 (10)	0.02208 (12)	−0.00018 (8)	0.00232 (8)	0.00028 (9)
Si1	0.0150 (5)	0.0213 (5)	0.0190 (5)	0.0031 (4)	0.0009 (4)	−0.0011 (4)
Si2	0.0214 (5)	0.0157 (5)	0.0236 (6)	−0.0009 (4)	−0.0052 (4)	0.0028 (4)
Si3	0.0177 (5)	0.0192 (5)	0.0191 (5)	−0.0009 (4)	0.0012 (4)	−0.0017 (4)
O1	0.0207 (14)	0.0355 (16)	0.0295 (16)	−0.0014 (12)	0.0062 (12)	−0.0009 (13)
C1	0.0112 (16)	0.0161 (17)	0.0209 (19)	0.0018 (14)	0.0018 (14)	0.0027 (15)
C2	0.0130 (17)	0.0140 (17)	0.025 (2)	0.0030 (14)	0.0021 (15)	0.0029 (15)
C3	0.0184 (18)	0.024 (2)	0.0162 (19)	0.0082 (15)	0.0003 (15)	0.0016 (15)
C4	0.0151 (17)	0.0177 (19)	0.025 (2)	0.0022 (14)	−0.0071 (15)	−0.0016 (16)
C5	0.0083 (16)	0.0175 (18)	0.030 (2)	−0.0002 (13)	0.0033 (15)	0.0049 (16)
C6	0.027 (2)	0.023 (2)	0.027 (2)	0.0011 (16)	−0.0006 (17)	−0.0044 (17)
C7	0.029 (2)	0.033 (2)	0.021 (2)	0.0055 (18)	−0.0059 (17)	0.0013 (17)
C8	0.023 (2)	0.031 (2)	0.024 (2)	0.0038 (17)	0.0045 (17)	−0.0018 (17)
C9	0.0162 (17)	0.0111 (17)	0.0212 (19)	0.0006 (13)	0.0003 (14)	0.0012 (14)
C10	0.0208 (18)	0.0103 (16)	0.0210 (19)	0.0006 (14)	0.0037 (15)	0.0040 (15)
C11	0.0133 (17)	0.0139 (17)	0.024 (2)	−0.0047 (13)	−0.0014 (15)	0.0047 (15)
C12	0.0241 (19)	0.0118 (17)	0.021 (2)	−0.0067 (15)	−0.0054 (15)	0.0020 (15)
C13	0.0208 (18)	0.0118 (17)	0.022 (2)	−0.0011 (14)	0.0012 (15)	0.0009 (15)
C14	0.056 (3)	0.020 (2)	0.037 (3)	0.004 (2)	−0.023 (2)	−0.0014 (19)
C15	0.022 (2)	0.049 (3)	0.043 (3)	0.0003 (19)	−0.0048 (19)	0.024 (2)
C16	0.028 (2)	0.0204 (19)	0.021 (2)	0.0025 (16)	−0.0007 (16)	0.0022 (16)
C17	0.0097 (16)	0.0196 (18)	0.0162 (19)	−0.0037 (14)	0.0024 (14)	0.0003 (15)
C18	0.0099 (16)	0.0156 (18)	0.025 (2)	−0.0029 (13)	0.0026 (14)	0.0024 (15)
C19	0.0129 (17)	0.028 (2)	0.0149 (19)	−0.0086 (15)	−0.0025 (14)	0.0029 (15)
C20	0.0165 (18)	0.0192 (19)	0.027 (2)	−0.0062 (15)	0.0096 (16)	−0.0061 (16)
C21	0.0124 (17)	0.0176 (18)	0.022 (2)	−0.0025 (14)	0.0044 (14)	0.0051 (15)
C22	0.056 (3)	0.026 (2)	0.023 (2)	−0.009 (2)	0.006 (2)	−0.0032 (18)
C23	0.031 (2)	0.029 (2)	0.024 (2)	−0.0056 (18)	0.0015 (18)	−0.0038 (17)
C24	0.028 (2)	0.044 (3)	0.048 (3)	0.008 (2)	0.002 (2)	−0.020 (2)
C25	0.034 (2)	0.030 (2)	0.028 (2)	−0.0032 (18)	−0.0060 (19)	0.0018 (18)
C26	0.042 (3)	0.032 (2)	0.028 (2)	0.006 (2)	0.012 (2)	−0.0031 (19)
C27	0.024 (2)	0.026 (2)	0.044 (3)	0.0008 (17)	0.0085 (19)	0.0089 (19)
C28	0.033 (2)	0.025 (2)	0.026 (2)	−0.0010 (17)	0.0071 (18)	0.0006 (17)

### Geometric parameters (Å, º)

**Table:**

Yb1—Cnt1	2.510	C7—H7B	0.9800
Yb1—Cnt2	2.513	C7—H7C	0.9800
Yb1—Cnt3	2.504	C8—H8A	0.9800
Cs1—Cnt1	3.197	C8—H8B	0.9800
Cs1—Cnt2	3.268	C8—H8C	0.9800
Cs1—Cnt3	3.159	C9—C13	1.424 (5)
Yb1—C12	2.742 (3)	C9—C10	1.434 (5)
Yb1—C21	2.750 (3)	C9—Cs1^iii^	3.587 (3)
Yb1—C20	2.757 (3)	C10—C11	1.398 (5)
Yb1—C13	2.775 (3)	C10—Cs1^iii^	3.559 (3)
Yb1—C3	2.777 (3)	C10—H10A	0.9500
Yb1—C4	2.778 (3)	C11—C12	1.410 (5)
Yb1—C5	2.778 (3)	C11—Cs1^iii^	3.427 (3)
Yb1—C11	2.779 (3)	C11—H11A	0.9500
Yb1—C17	2.785 (3)	C12—C13	1.411 (5)
Yb1—C2	2.787 (3)	C12—Cs1^iii^	3.379 (3)
Yb1—C19	2.787 (3)	C12—H12A	0.9500
Yb1—C1	2.788 (3)	C13—Cs1^iii^	3.457 (3)
Yb1—C18	2.802 (3)	C13—H13A	0.9500
Yb1—C10	2.802 (3)	C14—H14A	0.9800
Yb1—C9	2.832 (3)	C14—H14B	0.9800
Cs1—O1	3.095 (3)	C14—H14C	0.9800
Cs1—C2	3.309 (3)	C15—H15A	0.9800
Cs1—C21^i^	3.337 (3)	C15—H15B	0.9800
Cs1—C20^i^	3.367 (3)	C15—H15C	0.9800
Cs1—C12^ii^	3.379 (3)	C16—Cs1^iii^	3.810 (4)
Cs1—C17^i^	3.383 (3)	C16—H16A	0.9800
Cs1—C1	3.390 (3)	C16—H16B	0.9800
Cs1—C3	3.396 (3)	C16—H16C	0.9800
Cs1—C18^i^	3.401 (3)	C17—C21	1.417 (5)
Cs1—C19^i^	3.407 (3)	C17—C18	1.425 (5)
Cs1—C11^ii^	3.427 (3)	C17—Cs1^iv^	3.383 (3)
Cs1—C13^ii^	3.457 (3)	C18—C19	1.390 (5)
Cs1—C5	3.475 (3)	C18—Cs1^iv^	3.401 (3)
Cs1—C4	3.499 (3)	C18—H18A	0.9500
Cs1—C10^ii^	3.559 (3)	C19—C20	1.406 (5)
Cs1—C9^ii^	3.587 (3)	C19—Cs1^iv^	3.407 (3)
Si1—C1	1.854 (4)	C19—H19A	0.9500
Si1—C8	1.866 (4)	C20—C21	1.407 (5)
Si1—C7	1.866 (4)	C20—Cs1^iv^	3.367 (3)
Si1—C6	1.867 (4)	C20—H20A	0.9500
Si2—C9	1.848 (4)	C21—Cs1^iv^	3.337 (3)
Si2—C15	1.863 (4)	C21—H21A	0.9500
Si2—C16	1.867 (3)	C22—H22A	0.9800
Si2—C14	1.871 (4)	C22—H22B	0.9800
Si3—C17	1.856 (3)	C22—H22C	0.9800
Si3—C22	1.864 (4)	C23—H23A	0.9800
Si3—C24	1.865 (4)	C23—H23B	0.9800
Si3—C23	1.869 (4)	C23—H23C	0.9800
O1—C25	1.434 (5)	C24—H24A	0.9800
O1—C28	1.435 (4)	C24—H24B	0.9800
C1—C5	1.418 (5)	C24—H24C	0.9800
C1—C2	1.423 (5)	C25—C26	1.522 (5)
C2—C3	1.402 (5)	C25—H25A	0.9900
C2—H2A	0.9500	C25—H25B	0.9900
C3—C4	1.407 (5)	C26—C27	1.511 (6)
C3—H3A	0.9500	C26—H26A	0.9900
C4—C5	1.396 (5)	C26—H26B	0.9900
C4—H4A	0.9500	C27—C28	1.507 (5)
C5—H5A	0.9500	C27—H27A	0.9900
C6—H6A	0.9800	C27—H27B	0.9900
C6—H6B	0.9800	C28—H28A	0.9900
C6—H6C	0.9800	C28—H28B	0.9900
C7—H7A	0.9800		
			
Cnt1—Yb1—Cnt2	120.1	C15—Si2—C14	110.1 (2)
Cnt1—Yb1—Cnt3	116.6	C16—Si2—C14	105.67 (18)
Cnt2—Yb1—Cnt3	122.8	C17—Si3—C22	110.60 (16)
Cnt1—Cs1—O1	88.8	C17—Si3—C24	108.68 (17)
Cnt2—Cs1—O1	94.1	C22—Si3—C24	109.2 (2)
Cnt3—Cs1—O1	127.8	C17—Si3—C23	112.26 (16)
Cnt1—Cs1—Cnt3	109.0	C22—Si3—C23	107.77 (19)
Cnt1—Cs1—Cnt2	121.4	C24—Si3—C23	108.32 (19)
Cnt2—Cs1—Cnt3	114.3	C25—O1—C28	109.0 (3)
Yb1—Cnt1—Cs1	175.3	C25—O1—Cs1	132.2 (2)
Yb1—Cnt2—Cs1	172.5	C28—O1—Cs1	105.9 (2)
Yb1—Cnt3—Cs1	176.7	C5—C1—C2	105.4 (3)
C12—Yb1—C21	133.07 (10)	C5—C1—Si1	126.8 (3)
C12—Yb1—C20	148.28 (10)	C2—C1—Si1	127.7 (3)
C21—Yb1—C20	29.62 (10)	C5—C1—Yb1	74.82 (18)
C12—Yb1—C13	29.63 (10)	C2—C1—Yb1	75.16 (18)
C21—Yb1—C13	118.73 (10)	Si1—C1—Yb1	114.08 (15)
C20—Yb1—C13	121.02 (10)	C5—C1—Cs1	81.47 (19)
C12—Yb1—C3	106.89 (10)	C2—C1—Cs1	74.58 (18)
C21—Yb1—C3	75.43 (10)	Si1—C1—Cs1	111.25 (13)
C20—Yb1—C3	93.17 (11)	Yb1—C1—Cs1	134.51 (12)
C13—Yb1—C3	133.57 (10)	C3—C2—C1	109.1 (3)
C12—Yb1—C4	84.54 (10)	C3—C2—Yb1	75.02 (19)
C21—Yb1—C4	104.58 (10)	C1—C2—Yb1	75.27 (19)
C20—Yb1—C4	121.05 (10)	C3—C2—Cs1	81.44 (19)
C13—Yb1—C4	114.11 (10)	C1—C2—Cs1	80.94 (19)
C3—Yb1—C4	29.34 (10)	Yb1—C2—Cs1	138.44 (12)
C12—Yb1—C5	93.42 (10)	C3—C2—H2A	125.4
C21—Yb1—C5	116.94 (10)	C1—C2—H2A	125.4
C20—Yb1—C5	118.11 (10)	Yb1—C2—H2A	116.2
C13—Yb1—C5	120.08 (10)	Cs1—C2—H2A	105.3
C3—Yb1—C5	48.02 (10)	C2—C3—C4	108.0 (3)
C4—Yb1—C5	29.10 (10)	C2—C3—Yb1	75.79 (19)
C12—Yb1—C11	29.58 (10)	C4—C3—Yb1	75.34 (19)
C21—Yb1—C11	162.49 (10)	C2—C3—Cs1	74.48 (18)
C20—Yb1—C11	161.12 (11)	C4—C3—Cs1	82.34 (19)
C13—Yb1—C11	48.43 (10)	Yb1—C3—Cs1	134.71 (12)
C3—Yb1—C11	104.80 (10)	C2—C3—H3A	126.0
C4—Yb1—C11	76.00 (10)	C4—C3—H3A	126.0
C5—Yb1—C11	72.15 (10)	Yb1—C3—H3A	115.1
C12—Yb1—C17	104.81 (10)	Cs1—C3—H3A	110.0
C21—Yb1—C17	29.67 (9)	C5—C4—C3	107.5 (3)
C20—Yb1—C17	49.15 (10)	C5—C4—Yb1	75.45 (19)
C13—Yb1—C17	89.55 (10)	C3—C4—Yb1	75.31 (19)
C3—Yb1—C17	91.45 (10)	C5—C4—Cs1	77.52 (19)
C4—Yb1—C17	115.93 (10)	C3—C4—Cs1	74.17 (18)
C5—Yb1—C17	139.21 (10)	Yb1—C4—Cs1	130.28 (12)
C11—Yb1—C17	134.15 (10)	C5—C4—H4A	126.3
C12—Yb1—C2	132.66 (10)	C3—C4—H4A	126.3
C21—Yb1—C2	69.27 (10)	Yb1—C4—H4A	115.3
C20—Yb1—C2	74.47 (10)	Cs1—C4—H4A	114.4
C13—Yb1—C2	161.85 (10)	C4—C5—C1	109.9 (3)
C3—Yb1—C2	29.18 (10)	C4—C5—Yb1	75.44 (19)
C4—Yb1—C2	48.21 (10)	C1—C5—Yb1	75.66 (18)
C5—Yb1—C2	47.93 (10)	C4—C5—Cs1	79.4 (2)
C11—Yb1—C2	119.41 (10)	C1—C5—Cs1	74.73 (19)
C17—Yb1—C2	95.49 (10)	Yb1—C5—Cs1	131.24 (12)
C12—Yb1—C19	122.04 (10)	C4—C5—H5A	125.0
C21—Yb1—C19	48.35 (10)	C1—C5—H5A	125.0
C20—Yb1—C19	29.37 (10)	Yb1—C5—H5A	115.8
C13—Yb1—C19	92.85 (10)	Cs1—C5—H5A	112.9
C3—Yb1—C19	121.44 (10)	Si1—C6—H6A	109.5
C4—Yb1—C19	150.23 (10)	Si1—C6—H6B	109.5
C5—Yb1—C19	142.33 (10)	H6A—C6—H6B	109.5
C11—Yb1—C19	133.69 (10)	Si1—C6—H6C	109.5
C17—Yb1—C19	48.72 (10)	H6A—C6—H6C	109.5
C2—Yb1—C19	103.58 (10)	H6B—C6—H6C	109.5
C12—Yb1—C1	122.63 (10)	Si1—C7—H7A	109.5
C21—Yb1—C1	94.75 (10)	Si1—C7—H7B	109.5
C20—Yb1—C1	89.08 (10)	H7A—C7—H7B	109.5
C13—Yb1—C1	146.41 (10)	Si1—C7—H7C	109.5
C3—Yb1—C1	48.85 (10)	H7A—C7—H7C	109.5
C4—Yb1—C1	48.90 (10)	H7B—C7—H7C	109.5
C5—Yb1—C1	29.52 (9)	Si1—C8—H8A	109.5
C11—Yb1—C1	98.33 (10)	Si1—C8—H8B	109.5
C17—Yb1—C1	123.39 (10)	H8A—C8—H8B	109.5
C2—Yb1—C1	29.57 (9)	Si1—C8—H8C	109.5
C19—Yb1—C1	113.15 (10)	H8A—C8—H8C	109.5
C12—Yb1—C18	100.20 (10)	H8B—C8—H8C	109.5
C21—Yb1—C18	48.09 (10)	C13—C9—C10	105.1 (3)
C20—Yb1—C18	48.09 (10)	C13—C9—Si2	129.1 (3)
C13—Yb1—C18	74.83 (10)	C10—C9—Si2	124.3 (3)
C3—Yb1—C18	120.22 (10)	C13—C9—Yb1	73.08 (19)
C4—Yb1—C18	145.35 (10)	C10—C9—Yb1	74.11 (18)
C5—Yb1—C18	164.74 (10)	Si2—C9—Yb1	128.22 (15)
C11—Yb1—C18	123.02 (10)	C13—C9—Cs1^iii^	73.26 (18)
C17—Yb1—C18	29.55 (9)	C10—C9—Cs1^iii^	77.33 (18)
C2—Yb1—C18	116.83 (10)	Si2—C9—Cs1^iii^	104.19 (12)
C19—Yb1—C18	28.80 (10)	Yb1—C9—Cs1^iii^	127.59 (11)
C1—Yb1—C18	137.11 (10)	C11—C10—C9	109.8 (3)
C12—Yb1—C10	48.31 (10)	C11—C10—Yb1	74.58 (19)
C21—Yb1—C10	156.10 (10)	C9—C10—Yb1	76.40 (19)
C20—Yb1—C10	132.46 (11)	C11—C10—Cs1^iii^	73.21 (18)
C13—Yb1—C10	48.00 (10)	C9—C10—Cs1^iii^	79.51 (18)
C3—Yb1—C10	128.46 (10)	Yb1—C10—Cs1^iii^	129.76 (12)
C4—Yb1—C10	99.32 (10)	C11—C10—H10A	125.1
C5—Yb1—C10	84.76 (10)	C9—C10—H10A	125.1
C11—Yb1—C10	29.00 (10)	Yb1—C10—H10A	115.8
C17—Yb1—C10	134.24 (10)	Cs1^iii^—C10—H10A	114.2
C2—Yb1—C10	130.23 (10)	C10—C11—C12	107.9 (3)
C19—Yb1—C10	108.45 (10)	C10—C11—Yb1	76.42 (18)
C1—Yb1—C10	101.51 (10)	C12—C11—Yb1	73.76 (18)
C18—Yb1—C10	109.46 (10)	C10—C11—Cs1^iii^	83.81 (19)
C12—Yb1—C9	49.06 (10)	C12—C11—Cs1^iii^	76.14 (19)
C21—Yb1—C9	128.10 (10)	Yb1—C11—Cs1^iii^	136.45 (12)
C20—Yb1—C9	113.55 (10)	C10—C11—H11A	126.1
C13—Yb1—C9	29.39 (9)	C12—C11—H11A	126.1
C3—Yb1—C9	153.27 (10)	Yb1—C11—H11A	115.9
C4—Yb1—C9	124.75 (10)	Cs1^iii^—C11—H11A	107.2
C5—Yb1—C9	114.22 (10)	C11—C12—C13	107.7 (3)
C11—Yb1—C9	48.76 (10)	C11—C12—Yb1	76.66 (19)
C17—Yb1—C9	105.13 (10)	C13—C12—Yb1	76.46 (19)
C2—Yb1—C9	158.00 (10)	C11—C12—Cs1^iii^	79.95 (19)
C19—Yb1—C9	84.94 (10)	C13—C12—Cs1^iii^	81.20 (19)
C1—Yb1—C9	128.44 (10)	Yb1—C12—Cs1^iii^	140.67 (13)
C18—Yb1—C9	80.25 (10)	C11—C12—H12A	126.1
C10—Yb1—C9	29.49 (9)	C13—C12—H12A	126.1
O1—Cs1—C2	80.28 (8)	Yb1—C12—H12A	113.2
O1—Cs1—C21^i^	114.33 (8)	Cs1^iii^—C12—H12A	106.1
C2—Cs1—C21^i^	148.98 (8)	C12—C13—C9	109.6 (3)
O1—Cs1—C20^i^	138.03 (8)	C12—C13—Yb1	73.92 (19)
C2—Cs1—C20^i^	137.32 (9)	C9—C13—Yb1	77.53 (19)
C21^i^—Cs1—C20^i^	24.23 (8)	C12—C13—Cs1^iii^	75.02 (18)
O1—Cs1—C12^ii^	110.63 (8)	C9—C13—Cs1^iii^	83.51 (19)
C2—Cs1—C12^ii^	92.69 (8)	Yb1—C13—Cs1^iii^	135.26 (12)
C21^i^—Cs1—C12^ii^	105.96 (8)	C12—C13—H13A	125.2
C20^i^—Cs1—C12^ii^	89.07 (8)	C9—C13—H13A	125.2
O1—Cs1—C17^i^	107.40 (7)	Yb1—C13—H13A	115.3
C2—Cs1—C17^i^	127.34 (8)	Cs1^iii^—C13—H13A	108.8
C21^i^—Cs1—C17^i^	24.34 (8)	Si2—C14—H14A	109.5
C20^i^—Cs1—C17^i^	39.93 (8)	Si2—C14—H14B	109.5
C12^ii^—Cs1—C17^i^	128.47 (8)	H14A—C14—H14B	109.5
O1—Cs1—C1	104.30 (7)	Si2—C14—H14C	109.5
C2—Cs1—C1	24.49 (8)	H14A—C14—H14C	109.5
C21^i^—Cs1—C1	129.35 (8)	H14B—C14—H14C	109.5
C20^i^—Cs1—C1	113.14 (8)	Si2—C15—H15A	109.5
C12^ii^—Cs1—C1	88.68 (8)	Si2—C15—H15B	109.5
C17^i^—Cs1—C1	114.09 (8)	H15A—C15—H15B	109.5
O1—Cs1—C3	68.30 (8)	Si2—C15—H15C	109.5
C2—Cs1—C3	24.09 (8)	H15A—C15—H15C	109.5
C21^i^—Cs1—C3	133.69 (8)	H15B—C15—H15C	109.5
C20^i^—Cs1—C3	136.14 (9)	Si2—C16—Cs1^iii^	96.16 (13)
C12^ii^—Cs1—C3	116.22 (8)	Si2—C16—H16A	109.5
C17^i^—Cs1—C3	109.40 (8)	Cs1^iii^—C16—H16A	66.8
C1—Cs1—C3	39.65 (8)	Si2—C16—H16B	109.5
O1—Cs1—C18^i^	124.90 (8)	Cs1^iii^—C16—H16B	52.7
C2—Cs1—C18^i^	109.78 (8)	H16A—C16—H16B	109.5
C21^i^—Cs1—C18^i^	39.23 (8)	Si2—C16—H16C	109.5
C20^i^—Cs1—C18^i^	39.10 (8)	Cs1^iii^—C16—H16C	153.3
C12^ii^—Cs1—C18^i^	122.27 (8)	H16A—C16—H16C	109.5
C17^i^—Cs1—C18^i^	24.24 (8)	H16B—C16—H16C	109.5
C1—Cs1—C18^i^	91.83 (8)	C21—C17—C18	105.5 (3)
C3—Cs1—C18^i^	98.84 (8)	C21—C17—Si3	128.0 (3)
O1—Cs1—C19^i^	146.73 (8)	C18—C17—Si3	126.4 (3)
C2—Cs1—C19^i^	114.55 (8)	C21—C17—Yb1	73.79 (18)
C21^i^—Cs1—C19^i^	39.29 (8)	C18—C17—Yb1	75.89 (18)
C20^i^—Cs1—C19^i^	23.95 (8)	Si3—C17—Yb1	118.31 (14)
C12^ii^—Cs1—C19^i^	98.71 (9)	C21—C17—Cs1^iv^	75.99 (18)
C17^i^—Cs1—C19^i^	39.57 (8)	C18—C17—Cs1^iv^	78.58 (18)
C1—Cs1—C19^i^	91.36 (8)	Si3—C17—Cs1^iv^	108.65 (13)
C3—Cs1—C19^i^	112.73 (8)	Yb1—C17—Cs1^iv^	132.98 (11)
C18^i^—Cs1—C19^i^	23.56 (8)	C19—C18—C17	109.5 (3)
O1—Cs1—C11^ii^	87.61 (8)	C19—C18—Yb1	75.02 (19)
C2—Cs1—C11^ii^	82.35 (8)	C17—C18—Yb1	74.57 (18)
C21^i^—Cs1—C11^ii^	123.61 (8)	C19—C18—Cs1^iv^	78.45 (19)
C20^i^—Cs1—C11^ii^	111.22 (8)	C17—C18—Cs1^iv^	77.18 (18)
C12^ii^—Cs1—C11^ii^	23.90 (8)	Yb1—C18—Cs1^iv^	131.52 (11)
C17^i^—Cs1—C11^ii^	147.92 (8)	C19—C18—H18A	125.3
C1—Cs1—C11^ii^	88.14 (8)	C17—C18—H18A	125.3
C3—Cs1—C11^ii^	102.45 (8)	Yb1—C18—H18A	117.0
C18^i^—Cs1—C11^ii^	146.18 (8)	Cs1^iv^—C18—H18A	111.4
C19^i^—Cs1—C11^ii^	122.61 (8)	C18—C19—C20	108.2 (3)
O1—Cs1—C13^ii^	110.19 (8)	C18—C19—Yb1	76.18 (19)
C2—Cs1—C13^ii^	116.34 (8)	C20—C19—Yb1	74.12 (19)
C21^i^—Cs1—C13^ii^	85.36 (8)	C18—C19—Cs1^iv^	77.99 (19)
C20^i^—Cs1—C13^ii^	73.64 (8)	C20—C19—Cs1^iv^	76.46 (19)
C12^ii^—Cs1—C13^ii^	23.79 (8)	Yb1—C19—Cs1^iv^	131.87 (12)
C17^i^—Cs1—C13^ii^	109.44 (8)	C18—C19—H19A	125.9
C1—Cs1—C13^ii^	111.22 (8)	C20—C19—H19A	125.9
C3—Cs1—C13^ii^	139.38 (8)	Yb1—C19—H19A	115.9
C18^i^—Cs1—C13^ii^	111.98 (8)	Cs1^iv^—C19—H19A	112.2
C19^i^—Cs1—C13^ii^	90.34 (8)	C19—C20—C21	107.4 (3)
C11^ii^—Cs1—C13^ii^	38.65 (8)	C19—C20—Yb1	76.50 (19)
O1—Cs1—C5	105.94 (7)	C21—C20—Yb1	74.91 (19)
C2—Cs1—C5	38.83 (8)	C19—C20—Cs1^iv^	79.59 (19)
C21^i^—Cs1—C5	110.42 (8)	C21—C20—Cs1^iv^	76.66 (18)
C20^i^—Cs1—C5	101.17 (8)	Yb1—C20—Cs1^iv^	134.91 (12)
C12^ii^—Cs1—C5	109.56 (8)	C19—C20—H20A	126.3
C17^i^—Cs1—C5	91.53 (8)	C21—C20—H20A	126.3
C1—Cs1—C5	23.80 (8)	Yb1—C20—H20A	114.7
C3—Cs1—C5	38.38 (8)	Cs1^iv^—C20—H20A	110.3
C18^i^—Cs1—C5	71.25 (8)	C20—C21—C17	109.3 (3)
C19^i^—Cs1—C5	77.29 (8)	C20—C21—Yb1	75.48 (19)
C11^ii^—Cs1—C5	111.87 (8)	C17—C21—Yb1	76.54 (18)
C13^ii^—Cs1—C5	129.48 (8)	C20—C21—Cs1^iv^	79.11 (19)
O1—Cs1—C4	84.87 (8)	C17—C21—Cs1^iv^	79.67 (18)
C2—Cs1—C4	38.88 (8)	Yb1—C21—Cs1^iv^	136.70 (12)
C21^i^—Cs1—C4	112.41 (8)	C20—C21—H21A	125.3
C20^i^—Cs1—C4	112.73 (8)	C17—C21—H21A	125.3
C12^ii^—Cs1—C4	127.43 (8)	Yb1—C21—H21A	114.7
C17^i^—Cs1—C4	88.95 (8)	Cs1^iv^—C21—H21A	108.6
C1—Cs1—C4	39.03 (8)	Si3—C22—H22A	109.5
C3—Cs1—C4	23.49 (8)	Si3—C22—H22B	109.5
C18^i^—Cs1—C4	75.59 (8)	H22A—C22—H22B	109.5
C19^i^—Cs1—C4	89.67 (8)	Si3—C22—H22C	109.5
C11^ii^—Cs1—C4	121.18 (8)	H22A—C22—H22C	109.5
C13^ii^—Cs1—C4	150.24 (8)	H22B—C22—H22C	109.5
C5—Cs1—C4	23.09 (8)	Si3—C23—H23A	109.5
O1—Cs1—C10^ii^	74.63 (7)	Si3—C23—H23B	109.5
C2—Cs1—C10^ii^	98.69 (8)	H23A—C23—H23B	109.5
C21^i^—Cs1—C10^ii^	111.34 (8)	Si3—C23—H23C	109.5
C20^i^—Cs1—C10^ii^	108.30 (8)	H23A—C23—H23C	109.5
C12^ii^—Cs1—C10^ii^	38.08 (8)	H23B—C23—H23C	109.5
C17^i^—Cs1—C10^ii^	133.90 (8)	Si3—C24—H24A	109.5
C1—Cs1—C10^ii^	109.52 (8)	Si3—C24—H24B	109.5
C3—Cs1—C10^ii^	113.49 (8)	H24A—C24—H24B	109.5
C18^i^—Cs1—C10^ii^	147.28 (8)	Si3—C24—H24C	109.5
C19^i^—Cs1—C10^ii^	127.76 (8)	H24A—C24—H24C	109.5
C11^ii^—Cs1—C10^ii^	22.98 (8)	H24B—C24—H24C	109.5
C13^ii^—Cs1—C10^ii^	37.70 (8)	O1—C25—C26	105.8 (3)
C5—Cs1—C10^ii^	133.23 (8)	O1—C25—H25A	110.6
C4—Cs1—C10^ii^	136.14 (8)	C26—C25—H25A	110.6
O1—Cs1—C9^ii^	87.86 (7)	O1—C25—H25B	110.6
C2—Cs1—C9^ii^	120.23 (8)	C26—C25—H25B	110.6
C21^i^—Cs1—C9^ii^	88.67 (8)	H25A—C25—H25B	108.7
C20^i^—Cs1—C9^ii^	85.71 (8)	C27—C26—C25	101.6 (3)
C12^ii^—Cs1—C9^ii^	38.69 (8)	C27—C26—H26A	111.5
C17^i^—Cs1—C9^ii^	112.18 (8)	C25—C26—H26A	111.5
C1—Cs1—C9^ii^	125.32 (8)	C27—C26—H26B	111.5
C3—Cs1—C9^ii^	136.64 (8)	C25—C26—H26B	111.5
C18^i^—Cs1—C9^ii^	124.31 (8)	H26A—C26—H26B	109.3
C19^i^—Cs1—C9^ii^	106.95 (8)	C28—C27—C26	102.1 (3)
C11^ii^—Cs1—C9^ii^	38.49 (8)	C28—C27—H27A	111.3
C13^ii^—Cs1—C9^ii^	23.23 (8)	C26—C27—H27A	111.3
C5—Cs1—C9^ii^	147.92 (8)	C28—C27—H27B	111.3
C4—Cs1—C9^ii^	158.85 (8)	C26—C27—H27B	111.3
C10^ii^—Cs1—C9^ii^	23.16 (7)	H27A—C27—H27B	109.2
C1—Si1—C8	109.90 (16)	O1—C28—C27	106.8 (3)
C1—Si1—C7	109.33 (16)	O1—C28—Cs1	52.50 (16)
C8—Si1—C7	108.40 (17)	C27—C28—Cs1	137.3 (2)
C1—Si1—C6	110.42 (16)	O1—C28—H28A	110.4
C8—Si1—C6	110.06 (17)	C27—C28—H28A	110.4
C7—Si1—C6	108.70 (18)	Cs1—C28—H28A	112.0
C9—Si2—C15	112.34 (18)	O1—C28—H28B	110.4
C9—Si2—C16	108.64 (16)	C27—C28—H28B	110.4
C15—Si2—C16	107.99 (17)	Cs1—C28—H28B	60.1
C9—Si2—C14	111.75 (17)	H28A—C28—H28B	108.6

Symmetry codes: (i) *x*+1, *y*, *z*; (ii) −*x*+3/2, *y*−1/2, −*z*+1/2; (iii) −*x*+3/2, *y*+1/2, −*z*+1/2; (iv) *x*−1, *y*, *z*.

## References

[bb1] Ali, S. H., Deacon, G. B., Junk, P. C., Hamidi, S., Wiecko, M. & Wang, J. (2018). *Chem. Eur. J.* **24**, 230–242. 10.1002/chem.201704383 29057570

[bb2] Apostolidis, C. B., Deacon, G., Dornberger, E. T., Edelmann, F., Kanellakopulos, B., MacKinnon, P. & Stalke, D. (1997). *Chem. Commun.* pp. 1047–1048.

[bb3] Bel’sky, V. K., Gunko, Y. K., Bulychev, B. M., Sizov, A. I. & Soloveichik, G. L. (1990). *J. Organomet. Chem.* **390**, 35–44.

[bb4] Bruker (2013). *SAINT*. Bruker AXS Inc., Madison, Wisconsin, USA.

[bb5] Bruker (2014). *APEX2* and *SADABS*. Bruker AXS Inc., Madison, Wisconsin, USA.

[bb6] Evans, W. J. (2016). *Organometallics*, **35**, 3088–3100.

[bb7] Evans, W. J., Boyle, T. J. & Ziller, J. W. (1992). *Organometallics*, **11**, 3903–3907.

[bb8] Evans, W. J., Champagne, T. M., Davis, B. L., Allen, N. T., Nyce, G. W., Johnston, M. A., Lin, Y.-C., Khvostov, A. & Ziller, J. W. (2006). *J. Coord. Chem.* **59**, 1069–1087.

[bb9] Fieser, M. E., MacDonald, M. R., Krull, B. T., Bates, J. E., Ziller, J. W., Furche, F. & Evans, W. J. (2015). *J. Am. Chem. Soc.* **137**, 369–382. 10.1021/ja510831n 25541886

[bb10] González-del Moral, O., Martín, A., Mena, M., Morales-Varela, M. del C. & Santamaría, C. (2005). *Chem. Commun.* pp. 3682–3684. 10.1039/b504467g 16027910

[bb11] Groom, C. R., Bruno, I. J., Lightfoot, M. P. & Ward, S. C. (2016). *Acta Cryst.* B**72**, 171–179. 10.1107/S2052520616003954 PMC4822653 27048719

[bb12] Hitchcock, P. B., Lappert, M. F., Maron, L. & Protchenko, A. V. (2008). *Angew. Chem. Int. Ed.* **47**, 1488–1491. 10.1002/anie.200704887 18189261

[bb13] Huh, D. N., Ziller, J. W. & Evans, W. J. (2018). *Inorg. Chem.* **57**, 11809–11814. 10.1021/acs.inorgchem.8b01966 30182717

[bb14] MacDonald, M. R., Bates, J. E., Ziller, J. W., Furche, F. & Evans, W. J. (2013). *J. Am. Chem. Soc.* **135**, 9857–9868. 10.1021/ja403753j 23697603

[bb15] Mills, N. (2006). *J. Am. Chem. Soc.* **128**, 13649–13650.

[bb16] Morris, J. J., Noll, B. C., Honeyman, G. W., O’Hara, C. T., Kennedy, A. R., Mulvey, R. E. & Henderson, K. W. (2007). *Chem. Eur. J.* **13**, 4418–4432. 10.1002/chem.200700219 17455192

[bb17] Morss, L. R. (1976). *Chem. Rev.* **76**, 827–841.

[bb18] Palumbo, C. T., Darago, L. E., Windorff, C. J., Ziller, J. W. & Evans, W. J. (2018). *Organometallics*, **37**, 900–905.

[bb19] Rosiak, D., Okuniewski, A. & Chojnacki, J. (2018). *Polyhedron*, **146**, 35–41.

[bb20] Sheldrick, G. M. (2008). *Acta Cryst* A**64**, 112–122. 10.1107/S0108767307043930 18156677

[bb21] Sheldrick, G. M. (2015*a*). *Acta Cryst* A**71**, 3–8.

[bb22] Sheldrick, G. M. (2015*b*). *Acta Cryst.* C**71**, 3–8.

[bb23] Voskoboynikov, A. Z., Argarkov, A. Y., Shestakova, A. K., Beletskaya, I. P., Kuz’mina, L. O. & Howard, J. A. K. (1997). *J. Organomet. Chem.* **544**, 65–68.

